# Optimality and cooperativity in superselective surface binding by multivalent DNA nanostars†

**DOI:** 10.1039/d4sm00704b

**Published:** 2024-10-11

**Authors:** Christine Linne, Eva Heemskerk, Jos W. Zwanikken, Daniela J. Kraft, Liedewij Laan

**Affiliations:** a Department of Bionanoscience, TU Delft 2629 HZ Delft The Netherlands l.laan@tudelft.nl; b Soft Matter Physics, Huygens-Kamerlingh Onnes Laboratory, Leiden Institute of Physics 2300 RA Leiden The Netherlands kraft@Physics.LeidenUniv.nl

## Abstract

Weak multivalent interactions govern a large variety of biological processes like cell–cell adhesion and virus–host interactions. These systems distinguish sharply between surfaces based on receptor density, known as superselectivity. Present experimental studies typically involve tens or hundreds of interactions, resulting in a high entropic contribution leading to high selectivities. However, if, and if so how, systems with few ligands, such as multi-domain proteins and bacteriophages binding to their host, show superselective behavior is an open question. Here, we address this question with a multivalent experimental model system based on star shaped branched DNA nanostructures (DNA nanostars) with each branch featuring a single stranded overhang that binds to complementary receptors on a target surface. Each DNA nanostar possesses a fluorophore, to directly visualize DNA nanostar surface adsorption by total internal reflection fluorescence microscopy (TIRFM). We observe that DNA nanostars can bind superselectively to surfaces and bind optimally at a valency of three, for a given binding strength and concentration. We explain this optimum by extending the current theory with interactions between DNA nanostar binding sites (ligands). Our results add to the understanding of multivalent interactions, by identifying cooperative mechanisms that lead to optimal selectivity, and providing quantitative values for the relevant parameters. These findings inspire additional design rules which improve future work on selective targeting in directed drug delivery.

## Abstract 2

Our study shows that DNA nanostars with three binding sites (ligands) can (1) bind superselectively to surfaces based on receptor density, and (2) that interactions between ligands affect the optimum number of ligands required for superselectivity.

## Graphical abstract

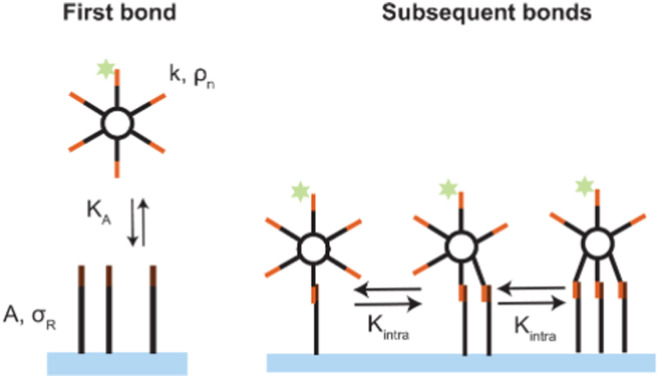

Multivalent interactions, where multiple ligands and receptors together form a single bond, are ubiquitous in nature. For example during bond formation by intrinsically disordered protein–protein interactions,^1–4^ ubiquitylation,^5^ antibody–antigen binding^6^ and virus–host binding,^7,8^ for example when a bacteriophage binds to specific receptors on the bacterial surface (Fig. 1a). In these examples individual ligand–receptor interactions are weak and highly reversible but together they establish a strong and often highly specific bond.

**Fig. 1:**
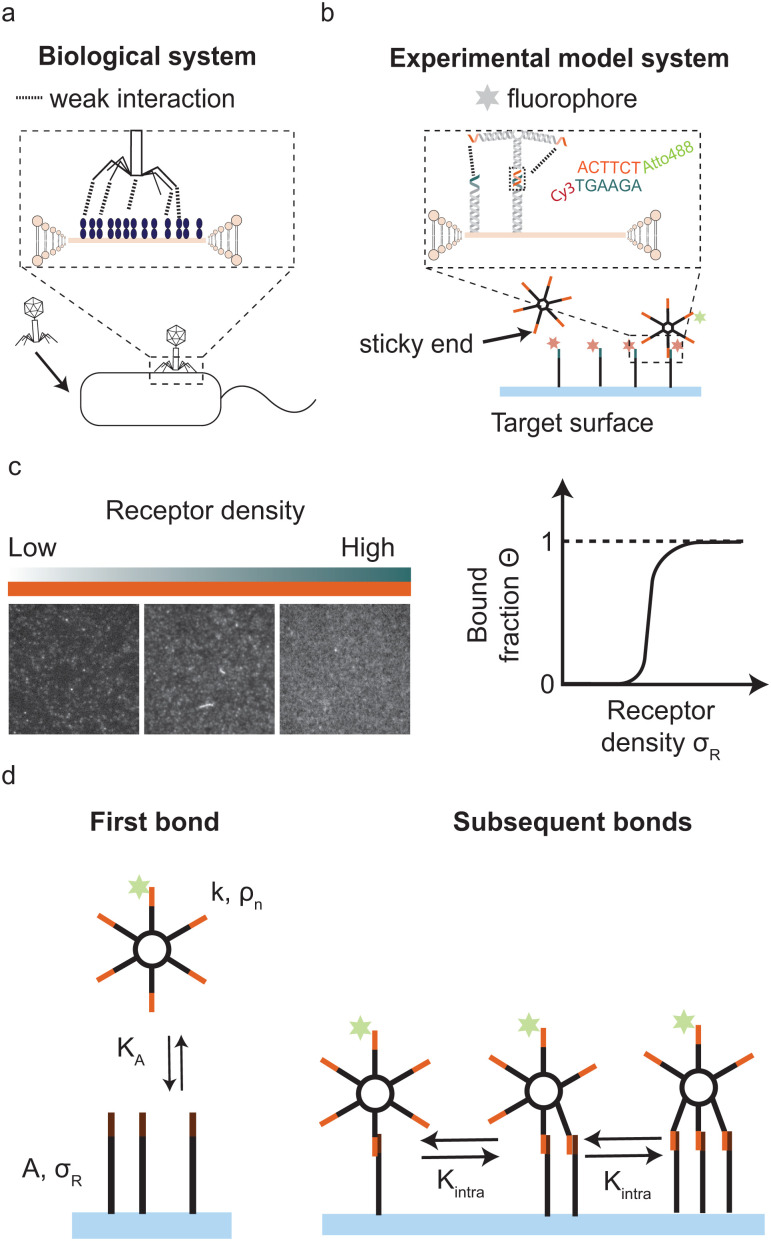
Motivation and model system (a) bacteriophage-host adhesion as an example where objects with <10 interaction sites participate in superselective surface binding. (b) DNA nanostars as a multivalent experimental model system for valencies below 10, that can bind to a supported lipid bilayer (SLB). Each arm of the DNA nanostar features a single stranded overhang (sticky end) that binds to the complementary sticky end on the receptors in the SLB. Each DNA nanostar possesses a fluorophore, such as Cy3 or Atto488, attached to one arm. (c) TIRFM images that show how the change in the number of adsorbed DNA nanostars on the target surface is quantified for variable number of receptors on the surface (see Methods section). The scalebar indicates 10 μm after background subtraction and normalisation with the saturated value, we translate the signal into a bound fraction *Θ* and plot it against the receptor density *σ*_R_ to determine the selectivity *α*. (d) Cartoon of the theory used to describe multivalent surface binding by DNA nanostars.

To understand how biological systems achieve high selectivity upon binding, Martinez-Veracoechea and Frenkel introduced the concept of superselectivity as a non-linear increase in the binding probability.^9^ In their model a multivalent particle distinguishes surfaces based on receptor density. A change in the interaction strength, valency and/or particle concentration manipulates the sharpness of this transition, where high valency, weak interactions and low particle concentrations yield the highest selectivity. In addition, recent studies illustrated that parameters like crowding,^10^ the addition of an external force on the particle^11^ and competition^12^ also regulate selectivity.

Present experimental systems that successfully demonstrated multivalent surface binding include polymers,^13,14^ viruses^7^ and nano-^15,16^ and microparticles.^17,18^ These systems either feature hundreds of interaction sites, as is the case for polymers and nano- and colloidal particles, or have limited experimental control over interaction strength and valency, as is the case for virus particles that interact with less than 10 receptors.^19^

In this paper we focus on multivalent surface binding by systems with few interaction sites, which occur, for example, during virus host binding,^7^ during binding of microtubules to chromosomes in mitosis^2^ or when multi-domain proteins bind to the cell membrane during polarity establishment in development.^20^ In addition, systems with few ligands can provide insights in the transition from monovalent to multivalent binding. Specifically we ask how selectivity of binding to receptor-covered surfaces by multivalent systems with few ligands depends on valency, interaction strength and physical properties of the system.

To experimentally address this question precise control is needed of valency, ligand and receptor interaction strength and the particle's concentration. In addition, control over other physical properties of this multivalent system, such as the flexibility of the ligands, self-interactions, and pair-interactions between the ligands is desirable for optimising the conditions for superselectivity. Here we exploit an experimental model system of DNA-origami nanostars to experimentally assess superselectivity in systems with low valencies and with tunable binding strength. A DNA nanostar consists of branched junctions of DNA strands also called arms with single stranded sticky overhangs that act as binding sites (ligands).^21–23^ The sticky ends on each DNA nanostar bind to surface mobile complementary DNA strands (receptors), see Fig. 1b. DNA nanostars have a number of attractive features: the length and sequence of the sticky end regulates the interaction strength, the number of arms precisely dictates the valency and a fluorophore attached to one arm allows for the visualization of DNA nanostar-surface adsorption with total internal reflection fluorescence microscopy (TIRFM).

We observe that multivalent DNA nanostars can bind superselectively to a surface coated with laterally mobile receptors, and we find that both valency and binding strength have an optimum for superselective surface binding. We extend the current theory by including interactions between DNA nanostars arms to be able to quantitatively match the observations, and find that the ligand pair-interaction strength has an optimum value for achieving maximal selectivity. From the extended model, we derive additional design rules for superselective surface binding and discuss what our findings could imply for biological systems.

## Results

### DNA nanostars as an experimental model system

Our experimental system to systematically study superselective surface targeting with 1–10 ligands consists of DNA nanostars. To quantitatively elucidate the transition of DNA nanostar adsorption, we employed DNA nanostars with different number of arms *k*, and imaged their adsorption to supported lipid bilayers (SLBs) functionalized with different receptor concentrations *σ*_R_. We employ DNA nanostars with a ssDNA sequence at the end of each arm (sticky end) that binds to receptors on a target surface with the complementary sticky end, see Fig. 1b. The receptors consist of two hybridized DNA strands that form a 77 bp double stranded stem and each feature a cholesterol moiety that together integrate the receptor into the SLB on the target surface while ensuring full mobility.^24–26^ On the 3′ end the receptors have a sticky end with the complementary sequence to the DNA nanostar sticky end. The length of the sticky end determines the hybridization free energy of each arm, see Materials and methods for details. Each DNA nanostar possesses an Atto488dye on the 3′ end of one arm, which does not inhibit binding.^27^ The excitation of the fluorophore and acquisition of the emission with total internal reflection microscopy (TIRFM) allows for the direct visualization of DNA nanostar-surface adsorption. The advantage of TIRFM is the direct excitation of the DNA nanostars on the surface and limited excitation of DNA nanostars in solution. We imaged the DNA nanostar signal for different receptor densities ranging from low to high, see (Fig. 1c). We measured the mean intensity of a certain area size, and normalised it with respect to the maximum intensity of the same area size. Finally, we plot the normalised signal, which is equal to the bound fraction *Θ* as a function of *σ*_R_, see (Fig. 1c). For details see the Materials and methods section.

### Optimal valency for superselective surface binding

**Fig. 2:**
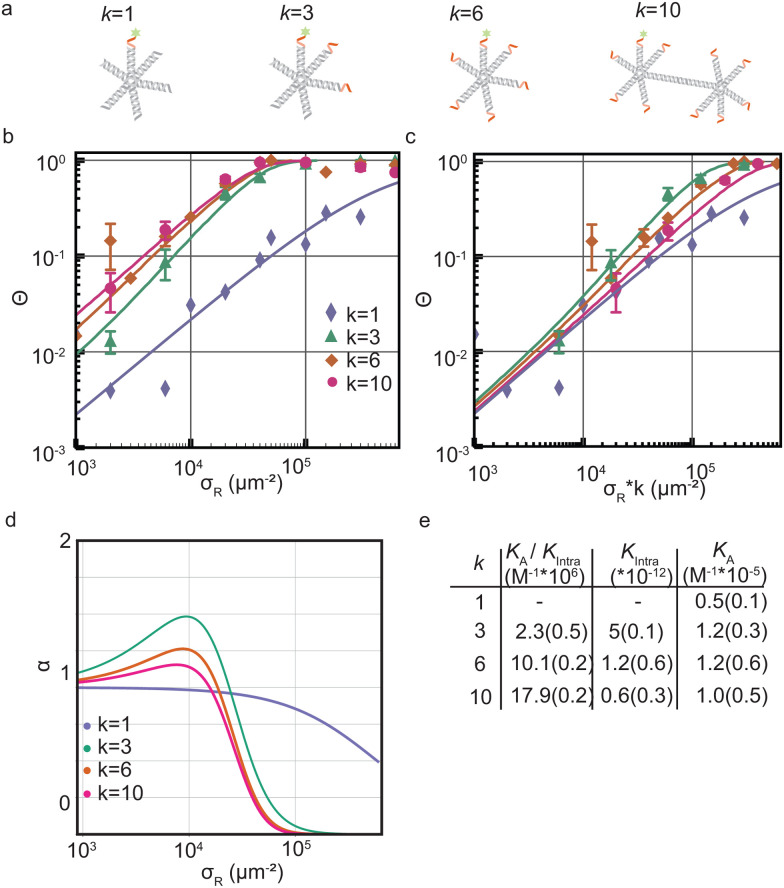
Optimality in valency of DNA nanostar surface binding (a) cartoons of DNA nanostars with a valency *k* of 1, 3, 6 or 10. The red parts indicate the single stranded binding sites. The fluorophore is depicted as green stars. (b) The bound fraction *Θ* measured as a function of receptor density *σ*_R_ for sticky end ACTTCT and four valencies *k* = 1,3,6,10. The lines are least-squared fits with eqn (3) adapted from Frenkel and coworkers^9^ with fitting parameters *K*^(0)^_A_ and *K*^(0)^_intra_. For *k* = 3 we excluded the last four datapoints for the fit to get the most accurate mathematical description of the non-linear transition of *Θ*. (c) The bound fraction *Θ* measured as a function of the receptor density *σ*_R_ rescaled with *k*. (d) The selectivity parameter *α* = d ln *Θ*/d ln *σ*_R_ as a function of receptor density *σ*_R_, shows an optimal *α* for *k* = 3. (e) Table with the bindings constants obtained from a fit with eqn (3) to *Θ* in (b) yields the fitting parameters *K*^(0)^_A_/*K*^(0)^_intra_ and *K*^(0)^_intra_. The numbers in brackets indicate the fitting error. A division of the two fitting parameters yields *K*^(0)^_A_.

We started by investigating the DNA nanostar-surface adsorption for different number of arms *k* = 1,3,4,10 but with fixed hybridization energy Δ*G*^0^ = −8.4*k*_B_*T* at a constant DNA nanostar concentration in solution *ρ*_n_ = 10^−8^ M (Fig. 2a). We choose this interaction strength based on our findings in a previous study with colloidal particles.^17^ We measured *Θ* over receptor densities ranging between *σ*_R_ = 1.000–600.000 μm^−2^, see Fig. 2b, and found that *Θ* smoothly increases with increasing *σ*_R_. With increasing valency *k* the curve shifts to lower *σ*_R_. This can be understood because DNA nanostars with a valency of *k* have a *k* times higher ligand concentration at the same concentration as a monovalent DNA nanostar. This would imply that the binding probability of the DNA nanostar scales linearly with *k*, if multivalent effects could be ignored.

To test this explanation for our experimental system, we multiply *σ*_R_ with *k*, see Fig. 2c, and we find that indeed the multivalent binding curves shift towards the monovalent curve and fall on top of each other in the low *σ*_R_ range. In this range, DNA nanostars most likely bind one arm only and thus effectively bind monovalently. However, at increasing *σ*_R_ the curves start to deviate from each other suggesting that the multivalent nature of the interaction causes a non-linear increase with *k*.

Next we determine how valency affects the selectivity of surface binding of DNA nanostars. The selectivity *α* of a multivalent system quantifies how sharp *Θ* increases with receptor density *σ*_R_:^9^1
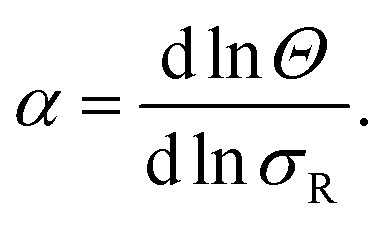
More precisely, *α* describes the slope of *Θ* as a function of *σ*_R_ on a log–log scale and *α* > 1 defines a superselective system. One can also interpret *α* as the (density-dependent) ‘Hill-coefficient’, with *Θ* ∝ *σ*^*α*^_R_ with a least-square fit of eqn (3) to the experimental data in Fig. 2b. Fig. 2d presents *α* as the derivative of the thus fitted curves for *Θ* in Fig. 2b. As expected for a monovalent DNA nanostar the selectivity never exceeds 1, because it follows the Langmuir isotherm, which has a maximum slope of 1. An increase in *k* is expected to lead to an increase in *α*. In Fig. 2d we indeed observe superselective behavior (*α* > 1) for our multivalent DNA nanostars and interestingly, we observe the largest selectivity for *k* = 3. Previous computational work on systems with many binding sites also showed an optimum in valency.^9^

To investigate the origin of this optimum, we determine the chemical equilibrium constants *K*^(0)^_A_ and *K*^(0)^_intra_ using the theory previously developed by Frenkel *et al.*^9^ (see also Fig. 1d). The superscript (0) indicates that the equilibrium constants are obtained *via* this model, treating the ligands as independent of each other. Their simplest model describes the adsorption of monovalent DNA nanostars as a Langmuir isotherm^9,28^ which is written in the specific form2
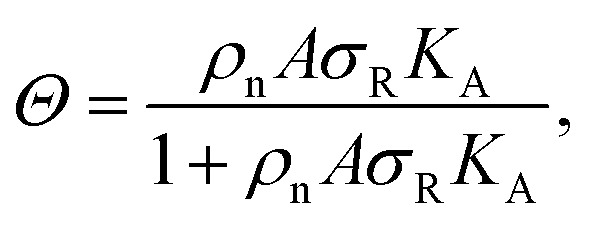
where *Θ* is the bound fraction, *ρ*_n_ is the DNA nanostar concentration in solution, *σ*_R_ is the receptor density on the target surface, *A* is a unit surface area and *K*_A_ is the equilibrium association constant to form a single bond, see Fig. 1e. The equilibrium constant *K*_A_ determines the specific concentration of receptors where half of the DNA nanostars are bound. In the expression we exchanged the activity *z* of the DNA nanostars by the concentration *ρ*_n_, given that the low concentrations in the experiments are in the nM-range.

The interaction strength between ligands and receptors shifts *Θ* relative to *σ*_R_, and larger interaction strengths (effectively described by *K*_A_) shift the transition point to lower concentrations. The number of arms *k* > 1 introduces a combinatorial term to the system that accounts for the number of possible bond formations, see Fig. 1d. The extra degrees of freedom of multivalent DNA nanostars add to specific entropy and energy differences between the bound states. Assuming that the arms act independently, and thus ignoring any (effective) attractions or repulsions between them, the adsorption can be written as3
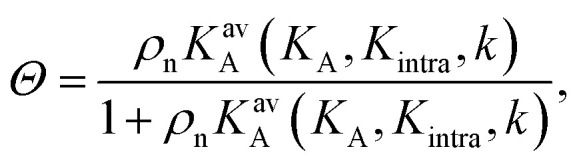
with *K*^av^_A_, the equilibrium avidity association constant, and *K*_intra_ is related to the equilibrium constant for the second bond, after the first bond is established (this constant would be (*k* − 1)*K*_intra_/2). The equilibrium constant *K*^av^_A_ is related to the formation of the first bond and, additionally, includes a combinatorial term that describes the formation of subsequent bonds:4
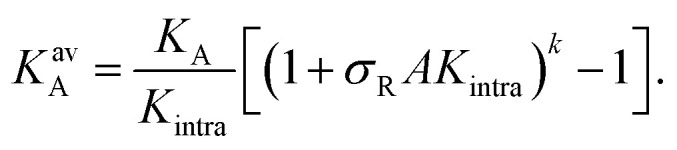
This equation approximately holds under appropriate conditions, far from the regime where saturation and surface receptor depletion occur.^9^ With stochastic simulations we explicitly tested whether these effects occurred and found that the number of available receptors is consistently close to the total number of receptors, thanks to the weak binding affinity of the DNA nanostars. Close to saturation where *Θ* ≈ 1 we did observe discrepancies between the data and eqn (3) as expected and incorporated that in the following fitting procedure by giving a larger weight to the low-coverage regime. The area *A* = 22 mm^2^ represents the total surface area of the flowcell, and defines the dimensionless unit of *K*_intra_.

As described above, if the bonds are formed independently of each other, and if the DNA nanostars effectively behave like monovalent particles, the binding probability of the multivalent particles scales with the valency *k*:

5 *Θ*_multi_ = *k* × *Θ*_mono_.

This would only be true if (1 + *σ*_R_*AK*_intra_)^*k*^ ≈ 1 + *kσ*_R_*AK*_intra_, and if *ρ*_n_*K*^av^_A_ ≪ 1 (*i.e.* far from the interesting superselective regime where multivalent interactions would be observable).

We used eqn (2) and (5) to fit the data. The comparison of *K*_A_ for *k* = 1,3,6,10 in Fig. 2e reveals no significant differences, consistent with the assumption we used in our model. Subsequent bond formations are captured by the second fitting parameter 1/*K*_intra_. Here in constrast, we observe a decrease in *K*_intra_ with increasing *k*. This suggests that DNA nanostars with more arms are less likely to bind with multiple arms to the SLB, which we will follow-up on later in this paper.

### Optimal binding strength for superselective surface binding

As a next step we studied the impact of interaction strength on superselectivity and the equilibrium binding constants. The model predicts that weakening the interaction strength will shift the curve of *Θ* towards larger *σ*_R_. The region where *Θ* ≪ 1 needs to be sufficiently large to facilitate a non-linear transition, which is determined by *K*_intra_.

**Fig. 3:**
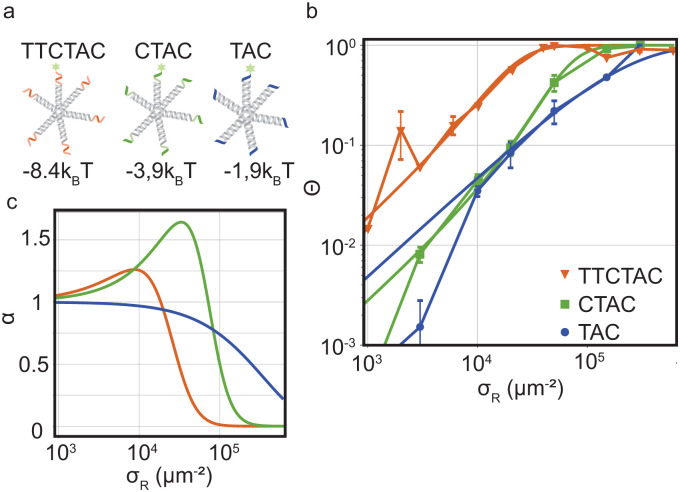
Optimality in binding strength of DNA nanostar surface binding (a) cartoons of DNA nanostars with varying binding strength. (b) The bound fraction *Θ* measured as a function of receptor density *σ*_R_ for nanostars with *k* = 6 and the three binding strength shown in (a). The lines are least-squared fits of the model eqn (3) adapted from Frenkel and coworkers^9^ with fitting parameters *K*_A_ and *K*_intra_. (c) The selectivity parameter 
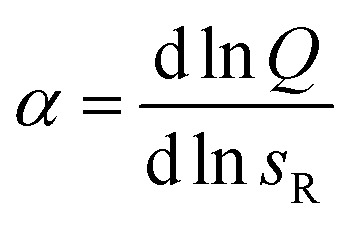
 as a function of receptor density *σ*_R_, shows an optimal *α* for a binding strength of −3.9*k*_B_*T*.

We test these theoretical predictions by measuring *Θ* for DNA nanostars with valency *k* = 6 and for three interaction strength of the individual arms, Δ*G*^0^ = −8.4*k*_B_*T* (sticky end sequence TTCTAC), −3.9*k*_B_*T* (CTAC) and −1.9*k*_B_*T* (TAC). The data of *Θ* as a function of *σ*_R_ for these DNA nanostars with three different sticky ends is presented in Fig. 3. Comparing the results, we immediately notice that *Θ*_TAC_ and *Θ*_CTAC_ shift to higher *σ*_R_ compared to *Θ*_TTCTAC_, in line with the theoretical predictions and with experiments performed on colloidal systems.^17^

To investigate if and how the selectivity and binding constants vary between these three nanostars, we fit eqn (3) on the data shown in Fig. 3b with *K*_A_ and *K*_intra_ as fitting parameters. We note that only the first four data points of the 4 bp sticky end were used in the fit to capture the non-linear transition as accurately as possible, because it determines the maximum selectivity of the system. We find that weakening the interaction strength from Δ*G*^0^ = −8.4*k*_B_*T* to −3.9*k*_B_*T* indeed makes the DNA nanostars more superselective. When we weaken the interaction strength even further to −1.9*k*_B_*T* the selectivity decreases again indicating that there is an optimal interaction strength to achieve highest superselectivity. Comparing the equilibrium constants *K*^(0)^_A_ and *K*^(0)^_intra_ of *k* = 6 for the 4 bp and 6 bp sticky ends (fits shown in Fig. 3b, and fit values reported in Table 1), we notice that whereas the equilibrium constant for binding the first arm, *K*^(0)^_A_, shows a difference of one order of magnitude, the values for the equilibrium constant associated to binding subsequent arms, *K*^(0)^_intra_, are similar for the two different sticky end lengths. The equilibrium constant *K*^(0)^_intra_ of the 3 bp sticky ends is much lower than that of the 4 bp sticky ends, while *K*_A_ is slightly larger. These are puzzling conclusions from the model fits, because one would expect *K*_A_ and *K*_intra_ to scale similarly with the binding strength, *K* ∝ exp(−*βf*), with *f* the binding free energy and *β* as 1/*k*_B_*T*. Presuming this ratio to be fixed did not yield meaningful fits and, together with the trends, indicates that the simplest version of the model is either incomplete, misses relevant interactions, or is inconsistent. One effect that is missing in the analytical model is the depletion of receptors,^29^ which were found to be relevant for our parameters *via* the simulation method (Fig. S3 in the ESI†). However, by fitting with the simulation data, we found that including this effect was not sufficient to mitigate the surprising observations described above.

**Table 1:** Bindings constants obtained from a fit to *Θ* in Fig. 3b with eqn (3) for the coverage of DNA nanostars with non-interacting ligands yields the fitting parameters *K*^(0)^_A_/*K*^(0)^_intra_ and *K*^(0)^_intra_. The numbers in brackets indicate the fitting error. A division of the two fitting parameters yields *K*^(0)^_A_, and the error is obtained through error propagation

Sticky end (bp)	*k*	*K* ^(0)^_A_/*K*^(0)^_intra_ (M^−1^ 10^7^)	*K* ^(0)^_intra_ (10^−13^)	*K* ^(0)^_A_ (M^−1^ 10^−5^)
TTCTAC	6	11.0(1.3)	12.3(21.3)	14.0(24)
CTAC	6	0.3(0.1)	6.0(1.0)	0.2(0.1)
TAC	6	13.0(7.0)	0.3(0.6)	0.4(0.8)

### Theory about optimum in valency and binding strength

The data in Fig. 2 demonstrate that a maximal selectivity is achieved for *k* = 3 arms, and suggest that *K*_intra_ is dependent on valency, given that the binding strength of the three different types of DNA nanostars was the same. To understand these puzzling observations it is helpful to consider the average number of bound arms of a bound DNA nanostar,6
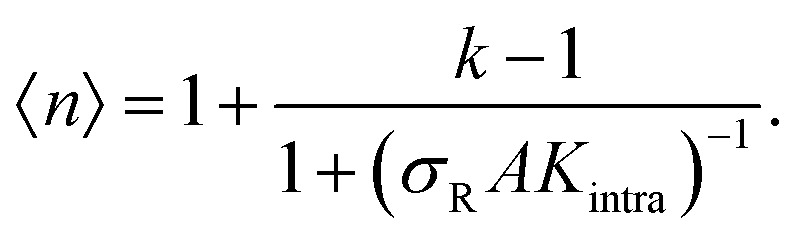
The number of bound arms, *n*, is effectively 1 if few receptors are available, and reaches the asymptote of *k* in the high *σ*_R_-limit. The derivation of this expression follows the same assumption as was used in eqn (3) (*i.e.* that the arms act independently), except that one only has to consider the microstates of a single DNA nanostar (more details are given in the ESI†). The receptor concentration where half of the arms are bound on average depends only on *K*_intra_ and not on the concentration of DNA nanostars. As long as 〈*n*〉 ≈ 1, the DNA nanostars behave effectively as monovalent particles, and the selectivity *α* ≈ 1. To a good approximation *α* ≈ 〈*n*〉, up to a point where *α* starts to drop to zero, because of saturation effects (of the surface, or depletion of the bulk), or to 1, if all available receptors become occupied. Whether a system behaves superselective or not, depends sensitively on this crossover density 
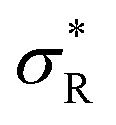
, with 
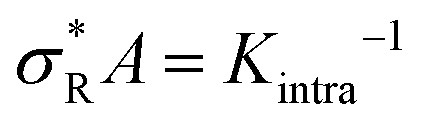
 (where the second term in eqn (6) starts to become significant). From this simple model, one can conclude that there are four distinct regimes, namely (1) for small 
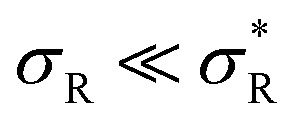
: particles bind with 1 arm on average and behave effectively monovalent, so *α* = 1, regime (2) around 
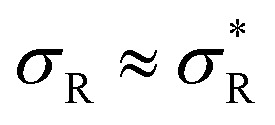
: the system becomes superselective, with *α* ≈ 〈*n*〉, regime (3) for higher 
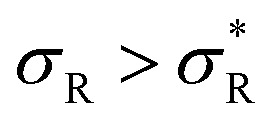
: *α* drops to 1 when all the available receptors become occupied, and regime (4) for high 
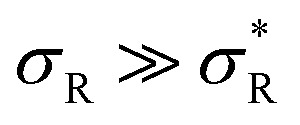
: *α* drops to zero due to saturation effects, which happens either when the bulk becomes depleted, or the surface becomes too crowded. Regime 1 and 4 should in principle always be achievable for any type of particle, but the interesting regime (2) is only visible if saturation effects are not important for 
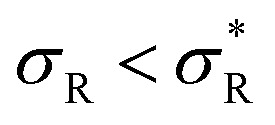
. This explains why a large number of arms *k* can be disadvantageous, because the larger *k* is, the sooner particles bind, and the sooner saturation effects appear. The density where the coverage is 50% will in general not coincide with the crossover density 
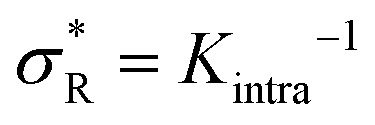
 where particles bind on average with 50% of their ligands, and occurs earlier with increasing *k*. Only if one can successfully prevent early binding *via* other principles such as steric repulsions and entropic barriers, could one achieve the maximum selectivity of *α* = *k*.

The analytical expressions contain in principle four tunable parameters: the two equilibrium constants *K*_A_ and *K*_intra_, the number of ligands *k*, and a maximum number of DNA nanostars per area *N*_max_ that determines the normalisation of *Θ*. The value of *N*_max_ determines when saturation effects become important, and is dependent on the size of the DNA nanostars, which set the maximum packing fraction. In scenarios with very low bulk concentrations, *N*_max_ is the available number of DNA nanostars in solution. The equilibrium constants *K*_intra_ and *K*_A_ are dependent on the binding strength of the ligands and their configurational degrees of freedom. Comparing now the binding probability of three different types of nanostars, with 3, 6 and 10 identical ligands, we would expect all parameters to be identical, except for the number of arms *k*. However, we observe (as described above) that the experimental data cannot be fitted with a single set of parameters, and only show reasonable agreement if we fit a different *K*_intra_-value to each set. This observation challenges the interpretation of *K*_intra_, and seems to be a clear indication that there are effects not captured by the model we used so far. Therefore we make a minimal extension of the model to estimate the potential influence of self-interactions and pair-interactions between the ligands.

**Fig. 4:**
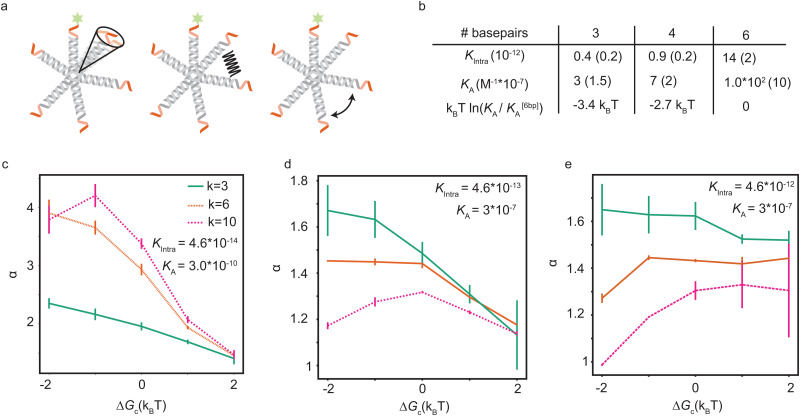
Model expansion with cooperative effects (a) to explain the experimentally observed effects, we include soft interactions between the ligands, for example due to steric or torsional effects between the arms or stiffness of the arms and joints, in our expanded model. These interactions may have an entropic or energetic origin. (b) Experimental fit values with the model which includes an interaction term for the equilibrium constants *K*_A_ and *K*_intra_. The ratio *K*_A_/*K*_intra_ was taken to be constant, because *K*_A_ and *K*_intra_ are expected to be equally affected by the binding strength. The difference in binding free energy relative to the 6 bp sticky end is given in the third row. (c)–(e) Simulation results show the maximal achievable selectivity as a function of pair-interaction strength Δ*G*_c_, which is ‘cooperative’ as Δ*G*_c_ < 0 and ‘competitive’ as Δ*G*_c_ > 0, for (c) *K*_A_ = 3.0 × 10^−10^ and *K*_intra_ = 4.6 × 10^−14^, (d) *K*_A_ = 3.0 × 10^−7^ and *K*_intra_ = 4.6 × 10^−13^ and (e) *K*_A_ = 3.0 × 10^−7^ and *K*_intra_ = 4.6 × 10^−12^ corresponding to the best fits with the experimental data. The equilibrium constants are highest in panel (e) (which corresponds best to the experimental data), and lowest in (c). There is an optimal Δ*G*_c_ corresponding to a weak cooperative interaction for small equilibrium constants, and a weak competitive interaction for higher values. The numerical error in *α* is negligible (<0.1%), but the estimation of the maximum *α* is based on a finite grid and introduces an uncertainty shown in the figures.

Soft pair interactions between the ligands and self-interactions could alter the binding kinetics, for example *via* steric interactions,^30^ ion-bridging, electrostatic interactions,^31–33^ or stiffness of the arms and joints^34,35^ (see Fig. 4a). The strength of these effects would depend on the total number of ligands *k*, being more important for particles with many ligands. Keeping the microscopic origin of these effects unspecified, we include a mesoscopic parameter Δ*G*_c_ to represent these effects in the form of a Gibbs free energy. We call the effect cooperative if Δ*G*_c_ < 0 and competitive if Δ*G*_c_ > 0. The situation where Δ*G*_c_ = 0 represents the original analytical model where the ligands act independently. This additional parameter enables connections between the mesoscopic phenomenon of cooperativity and the emergent selectivity. Although it will not clarify the microscopic origins of the cooperativity, which may be challenging to deconvolute, it represents a larger class of microscopic phenomena, leading to the same mesoscopic cooperativity. This parameter alters the transition rates between the states in the following way:
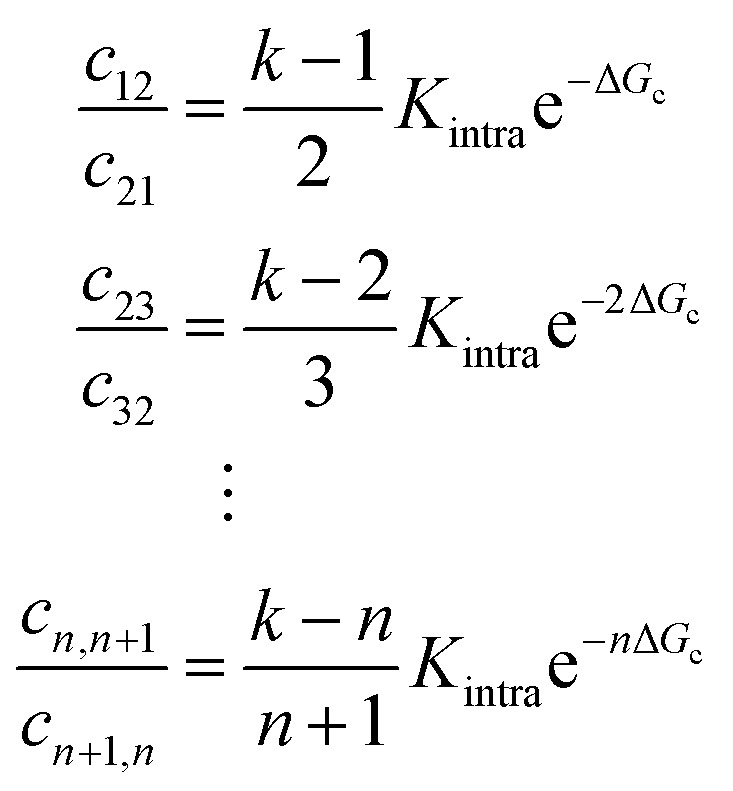
with *c*_*nm*_ the rate constant that a DNA nanostar makes a transition from a state with *n* arms bound to a state with *m* arms bound, with *m* = *n* ± 1. The combinatorial factor is the number of ligands available to bind, divided by the number of arms that are bound in state *n* + 1 and able to detach. The correction of the rate constant increases with *n*, representative of an attractive pair interaction, where *n* bound arms each interact with *n* − 1 other bound arms. In the simulations, we limited the maximal value of *n* to 4, representing a maximum number of neighbours that a ligand can interact with. However, this cutoff value was found not to influence the simulation results and fit values, because the fraction of particles binding with more than four arms was found to be completely negligible. This finding gives an indication for the low selectivity of the nanostars in our experiments.

Now we can fit this model to our experimental data to extract single values for *K*_A_ and *K*_intra_ from the experimental data, with a small difference in Δ*G*_c_, given in Table 2. The relative differences in Δ*G*_c_ indicate that the ligands of 3-armed DNA nanostars experience less competition than 10-armed DNA nanostars, with a difference of about 1.5*k*_B_*T* in the effective pair-interaction between the ligands.

**Table 2:** Parameter values that best fit the model to the experimental profiles. All three types of nanostars were fitted with *K*_intra_ = 14 × 10^−12^ and *K*_A_ = 1 × 10^−5^ M^−1^

# ligands (*k*)	3	6	10
Δ*G*_c_ (*k*_B_*T*)	−0.5	0	1.0

6-Armed DNA nanostars with 3 and 4 basepair sticky ends have a lower binding probability than those with 6 basepair sticky ends, as shown in Fig. 3. The length of the sticky end is expected to affect the unbinding rates^9^*k*_off_ ∝ exp(*βf*), with *f* the binding free energy of one ligand. Therefore the length of the sticky end is expected to influence *K*_intra_ and *K*_A_ by the same factor, as they both depend on 1/*k*_off_. Assuming that the pair interactions are not or only negligibly influenced by the sticky end, Δ*G*_c_ should be the same for these particles. Curve fits are found for the parameters given in the table of Fig. 4b. The difference in binding free energy between the different ligands is estimated by *k*_B_*T* ln(*K*_A_/*K*^[6bp]^_A_), relative to the binding free energy of the ligands with 6bp sticky ends.

The parameter values in the table of Fig. 4b suggest that a lower Δ*G*_c_, meaning more cooperation between the ligands, is favorable. However, a numerical exploration of parameter space seems to suggest that there is an optimal value of Δ*G*_c_, and that there are situations where competition between the ligands results in a higher selectivity than cooperation. To test this, we fit the experimental data using our expanded model. Fig. 4c–e show the maximal selectivity of a DNA nanostar as a function of Δ*G*_c_ for different values of the binding rates, being highest in the left figure and lowest in the right figure. Intuitively one expects cooperation to be favorable to achieve a higher selectivity, because it could make the average number of bound arms 〈*n*〉 depend more sensitively on the receptor density *σ*_R_ (eqn (6)), potentially even leading to a first order phase transition, where 〈*n*〉 makes a sudden jump at a critical value of *σ*_R_. Although this transition would drastically change the binding dynamics from solution, the availability of receptors would also drastically decrease, as the particles already bound would rapidly reduce the number of available receptors, and prevent particles from solution to bind. The effect of cooperation on the binding probability *Θ* is not as obvious as the effect on 〈*n*〉, and the simple relation between *α* and 〈*n*〉 that we found earlier does no longer hold in this scenario. For the parameter values that match the experimental data best, the results show that a small competition is actually favorable over a cooperative interaction, and that the maximal selectivity depends more sensitively on Δ*G*_c_ for DNA nanostars with more arms. The trivalent DNA nanostars have the highest selectivity and are least affected by the value of Δ*G*_c_. This observation suggests two important biological reasons why *e.g.* certain viruses, such as bacteriophages, would use a small number of ligands to select their host:^36–38^ (1) in a crowded environment, a smaller number of arms may result in a higher selectivity, and (2) the selectivity is stable under changing environmental conditions that influence the interactions between the arms.

## Discussion

Specific data would be required to interrogate the microscopic origin of the pair-interactions, and fix the zero-point of the Gibbs free energy Δ*G*_c_. As long as the DNA nanostars do not bind with more arms than 2, such that the selectivity *α* ≤ 2, the Gibbs free energy Δ*G*_c_ would simply rescale *K*_intra_, as is the case for our experimental results. In this case, there is effectively only one equilibrium constant between the first two bound states, which allows freedom where to set Δ*G*_c_ = 0 because there is only one transition with two variables, *K*_intra_ and Δ*G*_c_. Only in the parameter regime where *α* > 2 could one potentially validate the specific adaptation of the transition rates, and gain more information about the type of mechanism responsible for Δ*G*_c_. It is to be expected that the transition rates will depend on the number of bound arms, not only because of combinatorial reasons, but also because of the specific energy of the configuration, depending on the type of interaction. Data on the binding probability of particles with a selectivity *α* > 2 could shed light on the microscopic origin and strength of the pair-interactions. In the future it would be interesting to investigate DNA nanostar designs that are predicted to bind with a higher selectivity, for example by creating more flexibility within the DNA nanostar structure, or by introducing repulsive or attractive interactions between the DNA nanostar arms.

In summary, we have interrogated the effect of valency and binding strength on the selectivity of multivalent objects, with a limited valency *k* = 3, 6, 10, and found that both the valency and binding strength have an optimal value to achieve maximal selectivity. We observed that DNA nanostars with 3 ligands can be more selective than those with 6 and 10 ligands, and can explain this from the fact that particles with more ligands have a larger binding rate from solution, such that the surface saturates sooner, hindering binding in the superselective regime. After comparing the observations to the theoretical model, we also concluded that there may be relevant pair-interactions between the ligands. Including this effect at a mesoscopic level, we found agreement with our observations. By exploring parameter space with simulations, we found that the selectivity has a maximal value for an optimal strength of the cooperative interactions, and that there are even conditions where weak competitive interactions are optimal. These conditions include the parameters that were found from fits with the experimental data.

Based on our results we can formulate several design rules for maximizing selectivity under different experimental conditions: aligned with earlier conclusions,^9^ a maximal selectivity can be found at infinite dilution of the bulk solution, where *α* → *k*. In this limit, a larger valency always leads to a larger selectivity. However, this limit may not be experimentally accessible, as the equilibration time also drastically increases, and may not be relevant in a biological context, where concentrations are finite and selectivity needs to be established within a certain time interval. At finite concentrations, there is an optimal value of the valency and binding strength, which can be estimated with existing theoretical models, once the binding rates are measured. Weak interactions between the ligands complicate the picture, and have a larger influence the larger the valency is. Surprisingly, we find cooperative effects to be unfavorable for the experimental conditions, and only favorable if the on-rate from solution is sufficiently low, requiring a larger entropy barrier (by diluting the bulk solution) or stronger energy barrier (by *e.g.* steric hindrance) between the free and bound state. In conclusion, increasing the valency of a particle may actually lower the selectivity, and make the particles more sensitive to unwanted pair-interactions between the ligands. A limited valency may be favorable for maximizing the selectivity and robustness to environmental changes that affect the ligand–ligand interactions.

## Methods

### DNA nanostar hybridisation

All DNA strands were purchased from Integrated DNA Technologies Inc (IDT), resuspended in Tris buffer (pH8) and stored at −20 °C. To achieve for example tetravalent DNA nanostars with four sticky ends and one fluorophore, we mixed the four DNA strands *X*1, *X*2, *X*4 and *X*5 in equal molar ratios and annealed the mixture to 95 °C for 10 min and then cool it down at a rate of 0.2 °C min^−1^ to 4 °C, see Table S1 (ESI†). The annealing took place in a Thermocycler and a final concentration of 0.5 μM. The final product was stored at 4 °C. For the experiments, we diluted the desired concentration of DNA nanostars and receptors in Tris acetate–EDTA–NaCl (TAE, 100 mM NaCl, pH = 8) and 10 mM magnesium chloride (MgCl).

To verify the hybridization of the DNA nanostars, we performed DNA electrophoresis. The sample consisted of 10 μL of 0.5 μM DNA nanostars and we loaded the sample on a 1% agarose gel. After 30 min at 100 V we took an image of the gel, see Fig. S1 (ESI†). The fluorescent bands correspond to DNA nanostars: the higher the band, the larger the DNA nanostar nanostructure. Lower bands result from incomplete hybridizations. The intensity of the upper bands is significantly higher, confirming the successful formations of the DNA nanostars.

### DNA functionalised supported lipid bilayer

We studied the DNA nanostar adsorption in solution in a flow channel. The supported lipid bilayer (SLB) consisted of 18 : 1 1,2-dioleoyl-*sn-glycero*-3-phosphocholine (DOPC, [Avanti Polar lipids], stored in chloroform). To obtain the SLB, we first made small unilamellar vesicles (SUVs) from DOPC lipids. To do so, we added the desired volume of lipid to a glass vial and let it dry overnight in a vacuum desiccator. Subsequently, we resuspended the lipids in TAE–NaCl buffer and extruded the solution with an Avanti mini extruder through a membrane with pore size of 30 nm (Avanti Polar lipids). The microscopy slides and coverslips were sonicated at 30 min each in 2% Hellmanex solution, acetone (>99.9%) and potassium hydroxide solution (KOH, 1 M, [Merck]). Between each change of chemical we rinsed the glass ware [VWR] with milliQ water. Before use, the slides and coverglasses were blown dry with nitrogen. Parafilm stripes confined the flow channel and glued the microscopy slide and a coverslip together. Subsequent annealing at 125 °C let the Parafilm melt and bound the microscopy slide and coverslip together yielding (1 × 22) mm rectangular flow channels. To obtain SLBs in the flow channel, we injected SUVs and after 30 min at room temperature, we washed out the excess SUVs with buffer and added DNA of the desired concentration.

### Data acquisition and analysis

To image the DNA nanostar adsorption on the target surface we used total internal reflection microscopy (TIRF) on an inverted fluorescence microscope (Nikon Ti2-E) upgraded with an azimuthal TIRF/FRAP illumination module (Gata systems,iLAS 2) equipped with a 100× oil immersion objective (Nikon Apo TIRF, 1.49NA). Each DNA nanostar possesses an Atto488 dye and each receptor features a Cy3 dye. Therefore, we used laser excitations with wavelength 488 nm and 561 nm and detect the emitted fluorescent signal (EM-CCD Andor iXON Ultra 897). For each binding probability we measure for 7 different *σ*_R_ the intensity of the DNA nanostars *I* to obtain the full range of adsorption from unbound to bound. A negative control with *σ*_R_ = 0 μm^−2^ defines the background signal *I*_back_. The maximum intensity *I*_max_ provides a reference for normalization. The monovalent DNA nanostars were not measured until saturation due to practical constraints. Therefore, we normalized the monovalent signal with the maximum signal of *k* = 6 of the same sticky end. After the acquisition of the DNA nanostar adsorption in equilibrium, the acquired signal is corrected and normalized yielding the binding probability:7
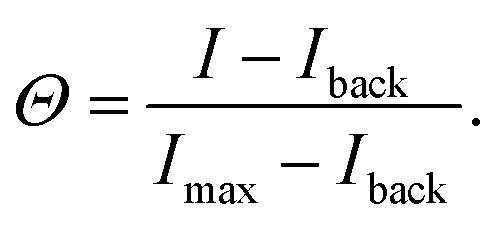
For the image processing we used a combination of ImageJ and python.

### Simulation method

The system is described as a reaction network, consisting of transitions between different bound states, and the free state in solution, with corresponding rate constants. This network is stochastically evolved using a kinetic Monte Carlo algorithm, according to Gillespie.^39^ After equilibration, the coverage is obtained as the average over a large number of iterations (*n* ≥ 10^5^). The binding probability was calculated by stochastically evolving a number of 10^6^ nanostars over the different binding states and free state according to the rate constants, and an explicit number of *N*_R_ receptors (per μm^2^). The maximal number of bound nanostars is estimated to be 10^4^ (per μm^2^), also informed by the observations, and fixed for all comparisons with experimental data. The other input parameters are the rate constants *k*_off_, representing the rate that a single specific ligand unbinds from a given receptor, *k*_on,sol_, representing the rate that a given ligand of a nanoparticle in solution binds to a given receptor, and *k*_on,sur_, representing the rate that a given ligand of a nanoparticle already bound to the surface binds to a given receptor. The value of *k*_off_ was fixed to be 1, basically defining the unit of time in the simulations. These three rate constants are converted to the experimental values according to8
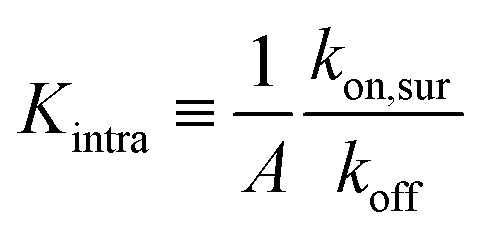
with *A* in μm^2^, and9
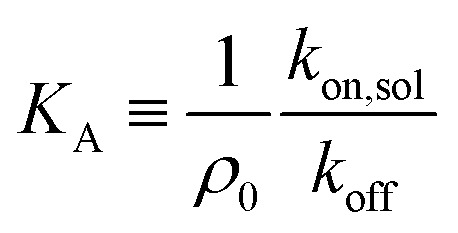
with *ρ*_0_ the concentration of nanoparticles in solution in *M*. To observe super selectivity, the rate constants needed to follow the hierarchy *k*_off_ ≫ *k*_on,sur_ ≫ *k*_on,sol_.

## Data availability

All data for this article, including experimental data of DNA nanostars binding to surfaces, (Fig. 1–3) and simulated data (Fig. 4) will be available at 4TU research.data upon acceptance for publication (https://data.4tu.nl).

## Conflicts of interest

There are no conflicts of interest to declare.

## Supplementary Material

SM-020-D4SM00704B-s001

## References

[cit1] Teilum K., Olsen J. G., Kragelund B. B. (2021). Biochem. J..

[cit2] Volkov V. A., Huis In’T Veld P. J., Dogterom M., Musacchio A. (2018). eLife.

[cit3] Maan R., Reese L., Volkov V. A., King M. R., van der Sluis E. O., Andrea N., Evers W. H., Jakobi A. J., Dogterom M. (2022). Nat. Cell Biol..

[cit4] Banjade S., Rosen M. K. (2014). eLife.

[cit5] Liu F., Walters K. J. (2010). Trends Biochem. Sci..

[cit6] Goldberg R. J. (2002). J. Am. Chem. Soc..

[cit7] Overeem N. J., Hamming P. H. E., Tieke M., van der Vries E., Huskens J. (2021). ACS Nano.

[cit8] Overeem N. J., Hamming P. H. E., Grant O. C., Iorio D. D., Tieke M., Bertolino M. C., Li Z., Vos G., de Vries R. P., Woods R. J., Tito N. B., Boons G.-J. P. H., van der Vries E., Huskens J. (2020). ACS Cent. Sci..

[cit9] Martinez-Veracoechea F. J., Frenkel D. (2011). Proc. Natl. Acad. Sci. U. S. A..

[cit10] Christy A. T. R., Kusumaatmaja H., Miller M. A. (2021). Phys. Rev. Lett..

[cit11] Curk T., Tito N. B. (2020). J. Phys.: Condens. Matter.

[cit12] Curk T., Dubacheva G. V., Brisson A. R., Richter R. P. (2022). J. Am. Chem. Soc..

[cit13] Dubacheva G. V., Curk T., Auzély-Velty R., Frenkel D., Richter R. P. (2015). Proc. Natl. Acad. Sci. U. S. A..

[cit14] Dubacheva G. V., Curk T., Mognetti B. M., Auzély-Velty R., Frenkel D., Richter R. P. (2014). J. Am. Chem. Soc..

[cit15] Lanfranco R., Jana P. K., Tunesi L., Cicuta P., Mognetti B. M., Di Michele L., Bruylants G. (2019). Langmuir.

[cit16] Phan H. T., Lauzon D., Vallée-Bélisle A., Angioletti-Uberti S., Chain J. L., Giasson S. (2023). Proc. Natl. Acad. Sci. U. S. A..

[cit17] Linne C., Visco D., Angioletti-Uberti S., Laan L., Kraft D. J. (2021). Proc. Natl. Acad. Sci. U. S. A..

[cit18] Scheepers M. R. W., van IJzendoorn L. J., Prins M. W. J. (2020). Proc. Natl. Acad. Sci. U. S. A..

[cit19] Szklarczyk O. M., González-Segredo N., Kukura P., Oppenheim A., Choquet D., Sandoghdar V., Helenius A., Sbalzarini I. F., Ewers H. (2013). PLoS Comput. Biol..

[cit20] Lang C. F., Munro E. M. (2022). Biophys. J..

[cit21] Conrad N., Kennedy T., Fygenson D. K., Saleh O. A. (2019). Proc. Natl. Acad. Sci. U. S. A..

[cit22] Brady R. A., Kaufhold W. T., Brooks N. J., Foderà V., Michele L. D. (2019). J. Phys.: Condens. Matter.

[cit23] Biffi S., Cerbino R., Bomboi F., Paraboschi E. M., Asselta R., Sciortino F., Bellini T. (2013). Proc. Natl. Acad. Sci. U. S. A..

[cit24] Van Der Meulen S. A., Dubacheva G. V., Dogterom M., Richter R. P., Leunissen M. E. (2014). Langmuir.

[cit25] Rinaldin M., Verweij R. W., Chakraborty I., Kraft D. J. (2019). Soft Matter.

[cit26] Chakraborty I., Meester V., van der Wel C., Kraft D. J. (2017). Nanoscale.

[cit27] van der Meulen S. A., Leunissen M. E. (2013). J. Am. Chem. Soc..

[cit28] Curk T. , Dobnikar J. and Frenkel D., Multivalency, John Wiley & Sons, Ltd, 2018, ch. 3, pp. 75–101

[cit29] Dubacheva G. V., Curk T., Frenkel D., Richter R. P. (2019). J. Am. Chem. Soc..

[cit30] Watzlawek M., Likos C. N., Löwen H. (1999). Phys. Rev. Lett..

[cit31] Raspaud E., De La Cruz M. O., Sikorav J.-L., Livolant F. (1998). Biophys. J..

[cit32] Sing C. E., Zwanikken J. W., Olvera de la Cruz M. (2013). Macromolecules.

[cit33] Randeria P. S., Jones M. R., Kohlstedt K. L., Banga R. J., Olvera de la Cruz M., Schatz G. C., Mirkin C. A. (2015). J. Am. Chem. Soc..

[cit34] Xing Z., Caciagli A., Cao T., Stoev I., Zupkauskas M., ONeill T., Wenzel T., Lamboll R., Liu D., Eiser E. (2018). Proc. Natl. Acad. Sci. U. S. A..

[cit35] Stoev I. D., Cao T., Caciagli A., Yu J., Ness C., Liu R., Ghosh R., ONeill T., Liu D., Eiser E. (2020). Soft Matter.

[cit36] Overeem N. J., Hamming P., Tieke M., Van Der Vries E., Huskens J. (2021). ACS Nano.

[cit37] Hussain W., Ullah M. W., Farooq U., Aziz A., Wang S. (2021). Biosens. Bioelectron..

[cit38] Singh A., Arya S. K., Glass N., Hanifi-Moghaddam P., Naidoo R., Szymanski C. M., Tanha J., Evoy S. (2010). Biosens. Bioelectron..

[cit39] Gillespie D. T. (1977). J. Phys. Chem..

