# Supplementary material for: Optimality and cooperativity in superselective surface binding by multivalent DNA nanostars
Source: Soft Matter. 2024 Oct 11;20(42):8515–23. doi: 10.1039/d4sm00704b (PMC11484159; doi:10.1039/d4sm00704b)
Supplement: SM-020-D4SM00704B-s001 [file SM-020-D4SM00704B-s001.pdf]

Supplementary Information (SI) for Soft Matter.
This journal is © The Royal Society of Chemistry 2024

Soft Matter

ARTICLE TYPE

Cite this: DOI: 00.0000/xxxxxxxxxx

Supplemental Information: Optimality and cooperativity in superselective surface binding by multivalent DNA nanostars

Christine Linne,[a,b] Eva Heemskerk,[a] Jos W. Zwanikken,[a] Daniela J. Kraft[b*], and Liedewij Laan[a*]

Received Date
Accepted Date

DOI: 00.0000/xxxxxxxxxx

[a] Department of Bionanoscience, TU Delft, 2629 HZ Delft, The Netherlands
[b] Soft Matter Physics, Huygens-Kamerlingh Onnes Laboratory, Leiden Institute of Physics,2300 RA Leiden, The Netherlands
∗ To whom correspondence should be addressed. E-mail: l.laan@tudelft.nl; kraft@Physics.LeidenUniv.nl

Derivation of the average number of bound ligands

Equation 6 in the manuscript describes the average number of bound ligands of a DNA nanostar, given that the particle is bound to the substrate (so that at least one ligand is bound).

The internal partition function of a single DNA nanostar, bound at the surface, is given by

$$Q = \sum_{m=0}^{k-1} \binom{k-1}{m} x^m, \tag{1}$$

with m representing the number of bound ligands beyond the first, and $x \equiv e^{\beta(\mu-\varepsilon)} = \sigma_R A K_{intra}$, using the analogy between the (equilibrium) rate constant K_{intra}, and potential energy difference ε between the unbound state and bound state of a ligand-receptor pair. For the chemical potential difference, we use the ideal gas approximation $\mu = k_B T \ln A\sigma_R$ which should break down at high densities of receptors, or when the number of free receptors is smaller than the total number of receptors, such that one can no longer speak of an effective 'reservoir' (with constant chemical potential) of receptors. However, this assumption holds well here because the particles bind weakly and reversibly (a condition for superselectivity) such that the number of free receptors is always close to the total number of receptors. The simulations work with explicit receptors and do not depend on this assumption, and confirm that the fraction of free receptors is larger than 0.95. Only for very superselective particles ($\alpha \geq 5$) did we observe a significant drop in the relative number of free receptors.

The expectation value $\langle n \rangle$ of the number of bound ligands, given that a particle is bound with at least one ligand, is now found by

$$\langle n \rangle = 1 + \frac{\sum_{m=0}^{k-1} m \binom{k-1}{m} x^m}{\sum_{m=0}^{k-1} \binom{k-1}{m} x^m}, \tag{2}$$

which is equal to

$$1 + \frac{\partial \ln Q}{\partial \ln x} = 1 + \frac{(k-1)x}{1+x}. \tag{3}$$

After substitution, one obtains equation 6 from the manuscript

$$\langle n \rangle = 1 + \frac{k-1}{1 + (\sigma_R A K_{intra})^{-1}} \tag{4}$$

This expression shows that the average number of bound arms is only dependent on K_{intra} and not on K_A. Furthermore, via simulations and an analytical derivation we found a useful approximation

$$\alpha \approx \langle n \rangle (1 - \Theta) \tag{5}$$

Therefore, one can interpret $\langle n \rangle$ as an effective Hill-coefficient, equal to α as long as there is no saturation. Hence, the rate constant K_{intra} determines the surface concentration where the particles start to bind with multiple arms (and become superselective), while the rate constant K_A needs to be sufficiently low, to delay saturation effects. These insights are based on the simple model, where the ligands act independently. We did observe that the convenient estimation, Equation 5, breaks down if $\Delta G_c \gg 1$, although not dramatically.

Methods

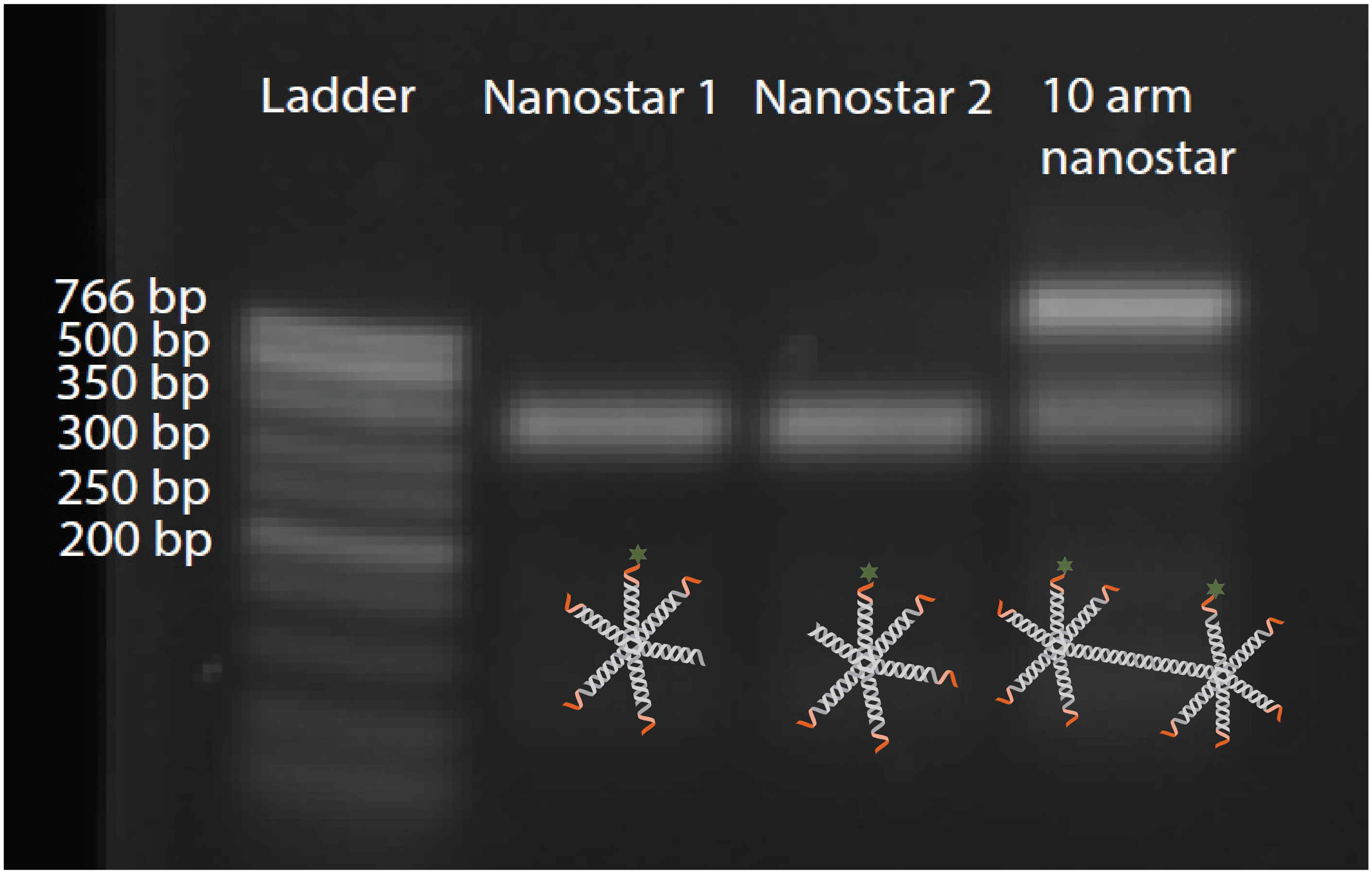

Fig. 1 DNA nanostar design DNA electrophoresis allows for the visualization of the final DNA star products after hybridization. The first lane shows the ladder. The second and third lane show the two different hybridized 6 arms nanostars that are subsequently hybridized together with a linker to form a 10 arm nanostar, which is shown in the fourth lane. The fluorescent bands shift upwards and confirm the formation of a larger DNA nanostructure. The lower band in the fourth lane shows there are some 6 arm DNA nanostars present but the majority is hybridized in a 10 arm DNA nanostar.

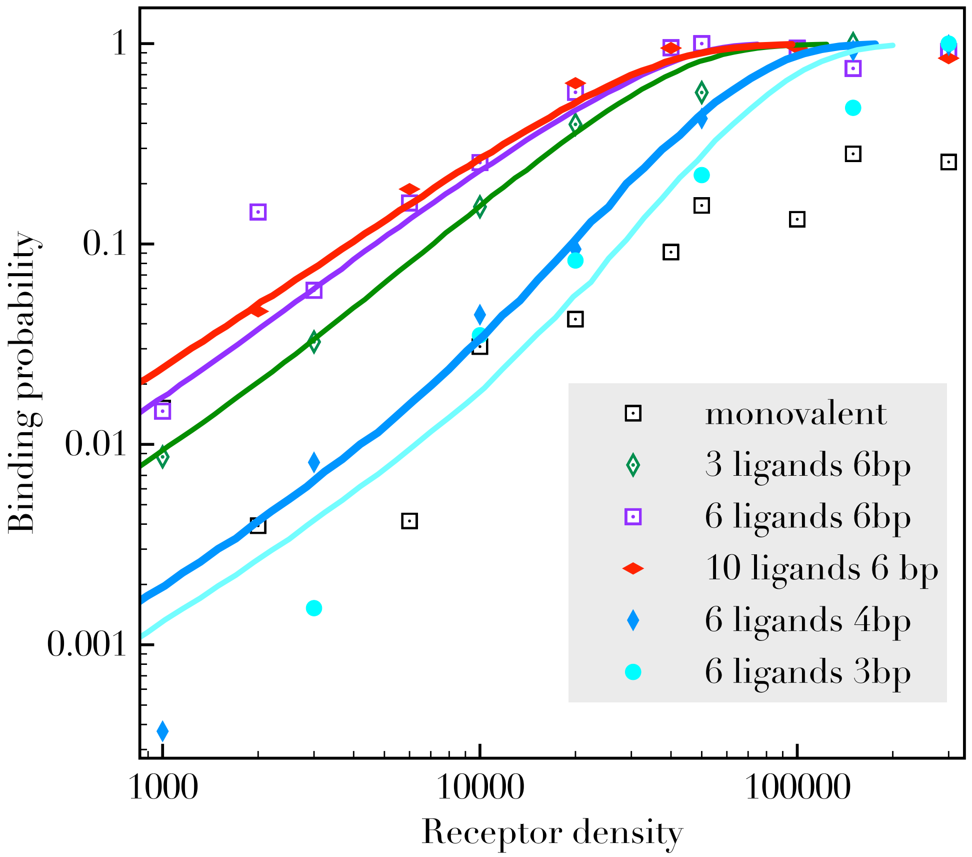

Fig. 2 The binding probability as a function of receptor density for three types of DNA nanostars with identical ligands (6 basepairs) but different valency (3, 6 and 10), and two types of nanostars with valency 6 and shorter sticky ends (3 and 4 basepairs). The symbols represent the experimental data and the curves are simulation results, using the same $K_{\rm intra}$ and $K_{\rm A} = 3 \cdot 10^{-5}$ M^{-1} for the curves with 6 bp, representing identical on- and off-rates, but with different $\Delta G_{\rm c}$, representing a different pair-interaction between the ligands. Best fits were obtained with values $K_{\rm intra} = 1.4 \cdot 10^{-11}$, $K_{\rm A} = 1 \cdot 10^{-6}$ M^{-1} and $\Delta G_{\rm c} = -0.5, 0, 1$ for the nanostars with 3, 6, and 10 ligands, respectively. The parameters would imply that DNA nanostars with a higher valency experience more competition in the binding process. The data of the nanostars with shorter ligands were fitted with the same $K_{\rm intra}/K_{\rm A}$ ratio, but with $K_{\rm intra} = 9 \cdot 10^{-12}$, $K_{\rm A} = 7 \cdot 10^{-7}$ M^{-1} (4 bp) and $K_{\rm intra} = 4 \cdot 10^{-12}$, $K_{\rm A} = 3 \cdot 10^{-7}$ M^{-1} (3 bp), while $\Delta G_{\rm c} = 0$ for all nanoparticles with 6 ligands.

Name	Sequence (5′ to 3′)	5′	3′
X 1	CTACTATGGCGGGTGATAAAAAAA CGGGAAGAGCATGCCCATCCA-*sticky end*	-	ATTO488
X 2	GGATGGGCATGCTCTTCCCGAAAA CTCAACTGCCTGGTGATACGA-*sticky end*	-	-
X 3	CGTATCACCAGGCAGTTGAGAAAA TTTATCACCCGCCATAGTAGA-*sticky end*	-	-
X 4	GTATCACCAGGCAGTTGAGAAAA CATGCGAGGGTCCAATACCGA-*sticky end*	-	-
X 5	CGGTATTGGACCCTCGCATGAAAA TTTATCACCCGCCATAGTAGA-*sticky end*	-	-
X 6	CGGTATTGGACCCTCGCATGAAAA CCATGCTGGACTCAACTGACA-*sticky end*	-	-
X 7	GTCAGTTGAGTCCAGCATGGAAAA TTTATCACCCGCCATAGTAGA-*sticky end*	-	-
X 8	GTCAGTTGAGTCCAGCATGGAAAA CGCATCAGTTGCGGCGCCGCA-*sticky end*	-	-
X 9	GCGGCGCCGCAACTGATGCGAAAA TTTATCACCCGCCATAGTAGA-*sticky end*	-	-
Inert strand A	CGTAAGGCAGGGCTCTCTAGATTGACTGTGCGAAGGGTAGCGATTTT	-	Cholesterol-TEG
Inert strand B	TTTATCGCTACCCTTCGCACAGTCAATCTAGAGAGCCCTGCCTTACGA	Cholesterol-TEG	-
receptor backbone	TCGTAAGGCAGGGCTCTCTAGACAGGGCTCTCTGAATGTGACTGTGC GAAGGTGACTGTGCGAAGGGTAGCGATTTT	-	Cholesterol-TEG
receptor	TTTATCGCTACCCTTCGCACAGTCACCTTCGCACAGTCA CATTCAGAGAGCCCTGTCTAGAGAGCCCTGCCTTACGA-*sticky end*	Cholesterol-TEG	Cy3
X *sticky end*	GTAG, GTGATT	-	-
receptor *sticky end*	CTAC, AATCAC	-	-
X bridge A	GGATGGGCATGCTCTTCCCGAAAACTCAACTGC CTGGTGATACGTCCACCAATCCACTAATCCT	-	-
X bridge B	GGATGGGCATGCTCTTCCCGAAAACTCAACTG CCTGGTGATACGAGGATTAGTGGATTGGTGGA	-	-

Table 1 DNA sequences The DNA strands labelled with X form the DNA nanostars. The number of strands and sticky ends define the geometry and valency of the nanostar. Inert strand A and inert strand B are the two components for the dsDNA used as inert strands. The hybridization of receptor backbone and receptor yields the receptor with a sticky end. To click two DNA nanostars together, the strands X bridge A and X bridge B in combination with the regular nanostar strands X generate a nanostar with valencies larger than 6.

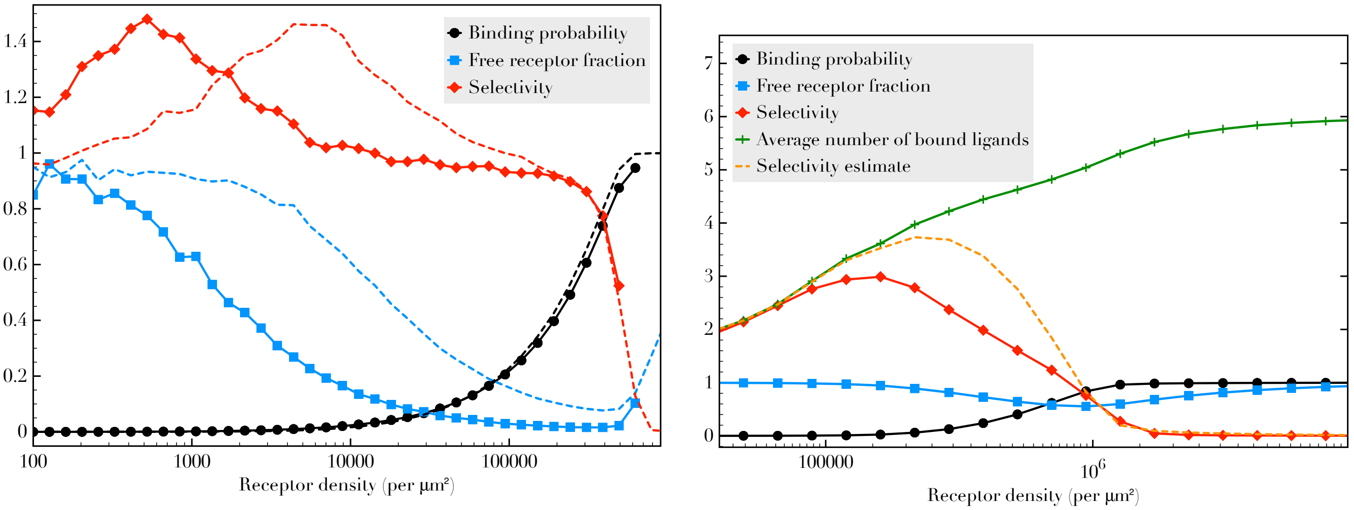

Fig. 3 Upper figure: Binding probability (black circle), fraction of receptors that is unbound (blue square) and selectivity (red diamond) as a function of the total receptor density, from stochastic simulations of the binding events. The dashed curves show the same quantities, but for a different K_{intra}, being $4.6\cdot10^{-10}$ for the continuous curves, and $4.6\cdot10^{-11}$ for the dashed curves. For these values $\sigma_{\mathrm{R}}AK_{\mathrm{intra}}=1$ if $\sigma_{\mathrm{R}}^{*}=10^{3}$ and 10^{4}, respectively, showing that the selectivity tends to peak at this receptor concentration (where the particles start binding with multiple arms). The constant $K_{\mathrm{A}}=3\cdot10^{-5}$ M^{-1}. These parameters corresponded best with the experimental data, except for the saturation point, which was observed around $\sigma_{\mathrm{R}}=10^{4}$. At this concentration, 60% of the receptors is unbound. The lower figure illustrates the relation between the selectivity and average number of bound arms, by showing the average number of bound ligands per bound nanostar (Equation 6 in the manuscript and 4 in this document) in green with $+$ symbols. In the lower figure $K_{\mathrm{intra}}=4.6\cdot10^{-12}$ and $K_{\mathrm{A}}=3\cdot10^{-7}$ M^{-1}. The orange dashed line is the estimated selectivity using $\alpha\approx\langle n\rangle(1-\Theta)$. This approximation is more accurate if the drop in selectivity is caused by the saturation of the surface (lower figure) rather than the relative depletion of receptors (upper figure). In the upper figure, the four binding regimes can be distinguished: 1) at low densities, the nanostars are not superselective and bind on average with one ligand 2) the superselective regime, where the nanostars start binding with multiple arms, $\sigma_{\mathrm{R}}AK_{\mathrm{intra}}\approx1$, 3) The regime where the selectivity drops due to nonlinear binding with receptors, and 4) the regime where the surface is fully covered or where all the nanostars are bound, where the selectivity drops to zero.
